# Alcohol consumption is associated with excessive risk of multiple sclerosis: a meta-analysis observational study

**DOI:** 10.1590/1516-3180.2021.0075.R1.14092021

**Published:** 2022-06-06

**Authors:** Haoyou Xu, Lijun Qiao, Supeng Fang, Zhanneng Ren, Guangliang Wu, Yu Zheng, Biying Yang, Yuanqi Zhao

**Affiliations:** I MD. Physician and Associate Professor, Department of Neurology, Guangdong Provincial Hospital of Chinese Medicine, Second Affiliated Hospital of Guangzhou University of Chinese Medicine, Guangzhou, China.; II PhD. Physician and Associate Professor, Department of Neurology, Guangdong Provincial Hospital of Chinese Medicine, Second Affiliated Hospital of Guangzhou University of Chinese Medicine, Guangzhou, China.; III BSc. Nurse, Department of Operating Theatre, Guangdong Provincial Hospital of Chinese Medicine, Second Affiliated Hospital of Guangzhou University of Chinese Medicine, Guangzhou, China.; IV MD. Physician and Assistant Professor, Department of Neurology, Guangdong Provincial Hospital of Chinese Medicine, Second Affiliated Hospital of Guangzhou University of Chinese Medicine, Guangzhou, China.; V PhD. Physician and Associate Professor, Department of Neurology, Guangdong Provincial Hospital of Chinese Medicine, Second Affiliated Hospital of Guangzhou University of Chinese Medicine, Guangzhou, China.; VI MD. Physician and Associate Professor, Department of Neurology, Guangdong Provincial Hospital of Chinese Medicine, Second Affiliated Hospital of Guangzhou University of Chinese Medicine, Guangzhou, China.; VII MD. Physician and Associate Professor, Department of Neurology, Guangdong Provincial Hospital of Chinese Medicine, Second Affiliated Hospital of Guangzhou University of Chinese Medicine, Guangzhou, China.; VIII PhD. Physician and Professor, Department of Neurology, Guangdong Provincial Hospital of Chinese Medicine, Second Affiliated Hospital of Guangzhou University of Chinese Medicine, Guangzhou, China.

**Keywords:** Alcohol drinking, Multiple sclerosis, Meta-analysis [publication type], Psychoneuroimmunology, Demyelinating diseases, Alcohol intake, Psychoimmunology, Demyelinating disorders

## Abstract

**BACKGROUND:**

There have been inconsistent results regarding the association between alcohol intake and susceptibility to multiple sclerosis.

**OBJECTIVE:**

To assess the potential role of alcohol intake regarding the risk of multiple sclerosis by using a meta-analytic approach.

**DESIGN AND SETTING:**

Observational meta-analysis study conducted in a hospital in China.

**METHODS:**

The electronic databases of PubMed, EMBASE and the Cochrane library were systematically searched for eligible studies from their inception up to January 2020. The summary odds ratio (OR) with 95% confidence interval (CI) was applied to assess the association between alcohol intake and multiple sclerosis, using a random-effects model.

**RESULTS:**

One prospective cohort study and eight case-control studies involving a total of 211,396 subjects and 10,407 cases of multiple sclerosis were selected for the final meta-analysis. From the pooled data, no significant association between alcohol intake and multiple sclerosis risk was found (OR: 0.94; 95% CI: 0.73-1.22; P = 0.668), and this conclusion was judged to be robust. Subgroup analysis found that intake of beer was associated with an increased risk of multiple sclerosis (OR: 1.58; 95% CI: 1.12-2.23; P = 0.010).

**CONCLUSION:**

This study found that beer intake could cause an excess risk of multiple sclerosis. Further large-scale prospective studies should be conducted to verify this conclusion.

## INTRODUCTION

Multiple sclerosis is an autoimmune disease of the central nervous system and is characterized by multifocal inflammatory demyelination and secondary axonal degeneration.^1^ It is a common neurological disorder and affects more than 2.3 million people worldwide. The most susceptible portion of the population is young adults.^2,3^ There is ample evidence to suggest that behaviorally and environmentally modifiable lifestyle factors could affect the progression, severity, symptoms and/or comorbidities of autoimmune diseases.^4,5^ Moreover, progression of multiple sclerosis might be balanced through changes to lifestyle, given that its progression involves inflammatory, metabolic and neurodegenerative disease processes.^6,7,8^

Studies have found that a healthy lifestyle could slow the progression and severity of multiple sclerosis. This indicates that a secondary prevention strategy should be applied to avoid deterioration due to multiple sclerosis.^9,10^ Smoking and alcohol intake have been identified as risk factors for autoimmune diseases. However, whether alcohol intake could affect the progression of multiple sclerosis remains a matter of debate.^11,12^ A study conducted by Hedström et al. found that alcohol intake presented a dose-dependent inverse association with multiple sclerosis.^13^ On the other hand, Massa et al. found that alcohol intake was not associated with the risk of multiple sclerosis.^14^

## OBJECTIVE

Clarifying the role of alcohol intake regarding the risk of multiple sclerosis is particularly important in the general population. We therefore attempted to undertake a comprehensive examination of published articles, in order to assess the association of alcohol intake with the risk of multiple sclerosis. Moreover, stratified analyses were also performed to assess whether the association between alcohol intake and multiple sclerosis might differ on the basis of study design, sex, type of alcohol or study quality.

## METHODS

### Data sources, search strategy and selection criteria

The Preferred Reporting Items for Systematic Reviews and Meta-Analysis (PRISMA) statement published in 2009 was used to guide the conduct of this meta-analysis.^15^ Eligible studies investigating the association between alcohol intake and multiple sclerosis risk were identified. The PubMed, EMBASE and Cochrane library databases were systematically searched to identify eligible studies up to January 2020, and the following core terms were applied: (“alcohol” or “beer” or “wine” or “hard liquor”) AND “multiple sclerosis”. The reference lists of relevant studies or reviews were also evaluated in order to select any new study that might meet the inclusion criteria.

The details of the inclusion criteria used in this study were as follows: 1) Study design: prospective cohort or case-control studies; 2) Exposure: alcohol intake, irrespective of the type of alcohol or alcohol dose; 3) Outcome: incidence of multiple sclerosis; and 4) the study needed to report on effect estimates, in order to make comparisons between high and low alcohol intake, regarding the risk of multiple sclerosis. The study selection process was conducted by two authors independently, and any conflicts were resolved through discussion with each other until a consensus was reached.

### Data collection and quality assessment

The abstracted information included the first author’s surname, publication year, country, study design, sample size, number of cases, age, percentage males, definition of alcohol intake, covariates in the full adjusted model and effect estimate with its 95% confidence interval (CI). Effect estimates that had been maximally adjusted for covariates were selected if the study reported several adjusted effect estimates. The quality of the studies included was assessed by using the Newcastle-Ottawa scale (NOS), which is based on selection (four items), comparability (one item) and outcome (three items), and the scoring system (expressed as a number of stars) ranged from 0 to 9 for each individual study.^16^

### Statistical analysis

The association between alcohol intake and the risk of multiple sclerosis was examined based on the effect estimate with 95% confidence interval (CI) for each study. The pooled odds ratio (OR) with 95% CI was then assessed using a random-effects model.^17,18^ Heterogeneity across the studies included was assessed using I^2^ and Q statistics. I^2^> 50.0% or P < 0.10 for the Q statistic was regarded as representing significant heterogeneity.^19,20^ The robustness of the pooled conclusion was assessed using sensitivity analysis, by means of sequentially excluding each study.^21^ Subgroup analyses were conducted based on study design, sex, alcohol type and study quality, and differences between groups were assessed using an interaction test.^22^ Publication bias was assessed by means of funnel plots, Egger tests and Begg tests.^23,24^ The inspection level for all pooled results was two-sided, and P < 0.05 was regarded as denoting a significant association between alcohol intake and multiple sclerosis risk. All statistical analyses in this meta-analysis were conducted using the Stata software (version 10.0; Stata Corporation, College Station, Texas, United States).

## RESULTS

### Literature search

**Figure 1.**
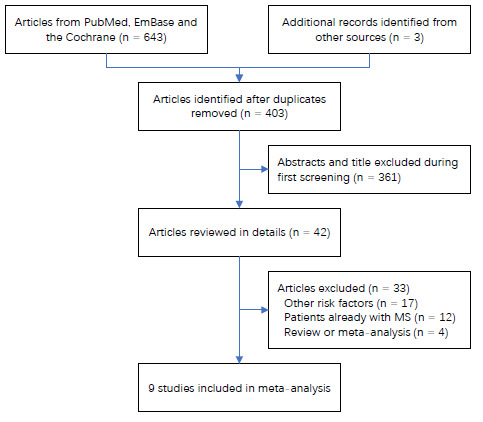
Flow diagram for the study selection process.

A total of 643 articles were identified from the initial electronic search, of which 240 were excluded because of duplicate titles. A further 361 studies were then excluded because of irrelevant titles. The remaining 42 studies were identified as meriting further full-text evaluations. Out of these, 33 studies were subsequently excluded, for the following reasons: other risk factors were addressed (n = 17); the patients already had multiple sclerosis (n = 12), whereas the aim of the present review was to assess the potential role of alcohol intake on the risk of multiple sclerosis and thus participants needed to be without this disease upon initial enrollment; or the study consisted of a review or meta-analysis (n = 4). No additional eligible study was found through reviewing the reference lists of the remaining studies that had been retrieved. In the end, nine studies that included a total of 211,396 subjects and 10,407 cases of multiple sclerosis were selected for the final meta-analysis.^13,14,25–31^ The details of the study selection are presented in Figure 1.

### Study characteristics

The baseline characteristics of the studies included and participants are summarized in Table 1. There was one study with a prospective cohort design, and the remaining eight studies were of case-control design. The sample size of the studies included ranged from 153 to 187,326, while 94 to 6,619 cases of multiple sclerosis were included in each study. Five studies were conducted in Europe, three studies were conducted in the United States or Canada, and the one remaining study was conducted in Iran. Study quality was assessed using the NOS: six studies had seven stars, two studies had six stars and the one remaining study had five stars.

**Table 1.** Baseline characteristics of studies included in the meta-analysis

Study	Country	Study design	Sample size	Number of cases	Age (years)	Percentage males (%)	Definition of alcohol intake	Adjusted factors	Study quality
Brosseau et al.^25^	Canada	Case-control	216	108	38.5	N/A	Self-administered questionnaire	Sex, age and same post-diagnostic period	5
Ghadirian et al.^26^	Canada	Case-control	399	197	39.0	31.3	Self-administered questionnaire	Age, sex and phone number	7
Pekmezovic et al.^27^	Serbia	Case-control	420	210	33.8	26.7	Self-administered questionnaire	Sex, age and residence in Belgrade district	6
Kotzamani et al.^28^	Greece	Case-control	1,250	657	43.6	38.2	Self-administered questionnaire	Age, sex and current residence	7
Massa et al.^14^	USA	Prospective cohort	187,326	258	25.0-55.0	0.0	Self-administered questionnaire	Age, total intake of vitamin D, residence at age 15 years, pack years of smoking and ethnicity	7
Hawkes et al.^29^	UK	Case-control	153	94	22.0-55.0	N/A	Self-administered questionnaire	Age of first symptoms, sex and smoking	6
Hedström et al.^13^	Sweden	Case-control cohort 1	2,506	745	16.0-70.0	24.8	Self-administered questionnaire	Age, sex and residential area	7
		Case-control cohort 2	11,120	5,874		26.9			
Abdollahpour et al.^30^	Iran	Case-control	1,604	547	31.0	41.1	Self-administered questionnaire	Age, sex, drug abuse, passive smoking, water-pipe smoking, tobacco smoking, sun exposure and current SES	7
Andersen et al.^31^	Denmark	Case-control	6,402	1,717	15.0-19.0	48.1	Self-administered questionnaire	Age, smoking at ages 15-19, body mass index at age 20, education and heredity.	7

N/A = not available; USA = United States of America; UK = United Kingdom; SES = socioeconomic status.

### Meta-analysis

**Figure 2.**
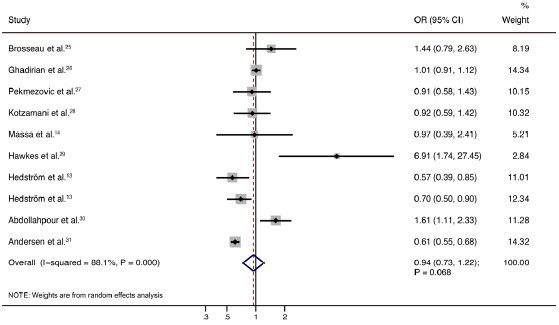
Association between alcohol intake and the risk of multiple sclerosis.

**Figure 3.**
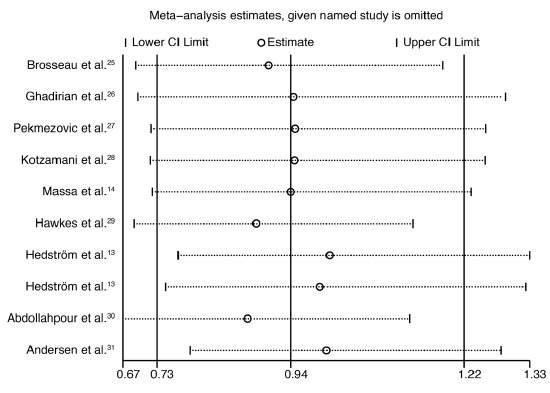
Sensitivity analysis on the association between alcohol intake and the risk of multiple sclerosis.

After pooling all the studies included, we noted that alcohol intake was not associated with the risk of multiple sclerosis (OR: 0.94; 95% CI: 0.73-1.22; P = 0.668; Figure 2). Moreover, significant heterogeneity was seen across these studies (I^2^= 88.1%; P < 0.001). The result from the sensitivity analysis suggested that it could be concluded that the data were robust and were not changed by sequentially excluding individual studies (Figure 3).

OR = odds ratio; CI = confidence interval.

CI = confidence interval.

### Subgroup analysis

Subgroup analysis on the association between alcohol intake and multiple sclerosis risk was conducted based on study design, sex, type of alcohol and study quality (Table 2). We noted that beer intake was associated with an increased risk of multiple sclerosis (OR: 1.58; 95% CI: 1.12-2.23; P = 0.010). Moreover, although no significant association between alcohol intake and multiple sclerosis risk was seen when males and females were analyzed together, there was a statistically significant difference between subgroups (P = 0.001). Similarly, although study quality could have affected the association between alcohol intake and multiple sclerosis risk, alcohol intake did not affect the risk of multiple sclerosis, irrespective of whether the pooled studies were of high quality or low quality (P = 0.022).

**Table 2.** Subgroup analysis on the association between alcohol intake and the risk of multiple sclerosis, based on study design, sex, type of alcohol and study quality

Factors	Subgroups	Number of studies	OR and 95% CI	P-value	Heterogeneity (I^2^)	P-value for ^I2^	P-value between subgroups
**Study design**	Prospective cohort	1	0.97 (0.39-2.41)	0.948	–	–	0.700
	Case-control	8	0.94 (0.72-1.24)	0.679	89.4	< 0.001	
**Sex**	Male	3	1.21 (0.89-1.63)	0.223	80.2	< 0.001	0.001
	Female	4	0.94 (0.67-1.34)	0.742	90.5	< 0.001	
**Type of alcohol**	Liquor	2	1.33 (0.38-4.74)	0.656	89.0	0.003	0.758
	Beer	2	1.58 (1.12-2.23)	0.010	0.0	0.621	
	Wine	2	1.41 (0.77-2.57)	0.270	54.1	0.140	
**Study quality**	High	6	0.85 (0.64-1.12)	0.241	90.4	< 0.001	0.022
	Low	3	1.63 (0.72-3.69)	0.239	74.9	0.018	

OR = odds ratio; CI = confidence interval.

### Publication bias

**Figure 4.**
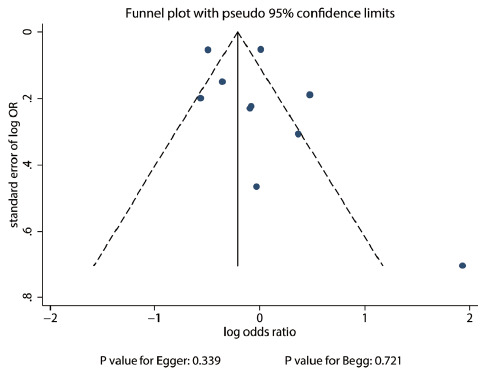
Publication bias for the association between alcohol intake and the risk of multiple sclerosis.

The publication bias regarding the association of alcohol intake with the risk of multiple sclerosis is displayed in Figure 4. No significant publication bias was detected by using the Egger test (P = 0.339) or Begg test (P = 0.721).

OR = odds ratio.

## DISCUSSION

The symptoms of multiple sclerosis are wide-ranging, including visual impairment, muscle weakness, sensory impairment and pain, which may be associated with increased risk of mood disorders and suicidal ideation. Whether drinking habits could affect the risk of multiple sclerosis has not been well studied, although the symptoms of multiple sclerosis are often correlated with alcohol disorders. Our meta-analysis was conducted on the basis of published articles and explored any potential role that alcohol intake might have in relation to subsequent multiple sclerosis risk. This study combined a total of 211,396 subjects and 10,407 cases of multiple sclerosis from one prospective cohort study and eight case-control studies across a wide range of characteristics among these individuals. We found that alcohol intake was not associated with the risk of multiple sclerosis, and this conclusion was relatively stable. Moreover, subgroup analysis found that beer intake was associated with an excess risk of multiple sclerosis.

A previous meta-analysis on seven studies found that alcohol intake was not associated with the risk of multiple sclerosis, and that conclusion was not altered by using sensitivity and subgroup analysis.^32^ Moreover, it was found that alcohol intake might protect against the risk of multiple sclerosis in the general population, while it caused a harmful effect on the risk of multiple sclerosis among people with other diseases. However, these effects were not associated with statistically significant differences.

Nevertheless, additional new published studies should be entered into the meta-analysis, and the conclusion regarding the association between alcohol intake and multiple sclerosis risk needs to be updated. Furthermore, there is a need to explore whether the type of alcohol intake yields different results in relation to the risk of multiple sclerosis. Hence, the current updated meta-analysis was conducted to systematically assess the role of alcohol intake and subsequent multiple sclerosis risk.

There was no significant association between alcohol intake and the risk of multiple sclerosis when all of the studies included were pooled. Although most of the studies included reported similar conclusions, it has been found in several other studies that alcohol intake may present beneficial or harmful effects regarding the risk of multiple sclerosis. Hawkes et al. found that alcohol intake was associated with an increased risk of multiple sclerosis after adjustments for the age at which the first symptoms appeared, sex and smoking.^29^ However, that study had a smaller sample size and included cases of pre-existing multiple sclerosis, and the result was not robust. Hedström et al. found that alcohol intake displayed a dose-dependent inverse relationship regarding the risk of multiple sclerosis. Moreover, alcohol intake could balance the potential role of smoking in relation to the risk of multiple sclerosis.^13^ In addition, Abdollahpour et al. suggested that alcohol intake was significantly associated with an increased risk of multiple sclerosis.^30^ Lastly, Andersen et al. suggested that alcohol intake during adolescence plays a protective role regarding the risk of multiple sclerosis, irrespectively for males or females.^31^ The potential reason for this could be that alcohol might have a dose-dependent immunomodulatory property.^33,34^ Alcohol could cross the blood-brain barrier and affect the immune and nervous systems.

Through subgroup analysis, it was found that beer intake might have a harmful effect regarding the risk of multiple sclerosis. This result may be explained by the findings from the study conducted by Abdollahpour et al., in which it was found that alcohol intake was associated with an increased risk of multiple sclerosis, irrespective of the type of alcohol.^30^ On the other hand, this result was calculated on the basis of only two studies and the pooled conclusion was variable. Furthermore, we noted that the potential role of alcohol intake regarding the risk of multiple sclerosis differed between males and females, although the protective or harmful effect trends were not associated with any statistically significant difference. In addition, the association between alcohol intake and the risk of multiple sclerosis could be affected by study quality. Potential differences in this regard might be correlated with immunomodulatory properties, dose of alcohol intake and the evidence level of published articles.

Several limitations of this meta-analysis need to be acknowledged: 1) the analysis contained both prospective and retrospective observational studies, and the results may have been affected by selection, recall and confounder biases; 2) the dose-response relationship for the role of alcohol intake in relation to multiple sclerosis risk was not investigated because the analysis needed restricted cubic splines with three knots at fixed percentiles of 10%, 50%, and 90% of the distribution, which was not available from the studies included; 3) the adjusted covariates across the studies included were different, which could have affected the association between alcohol intake and the risk of multiple sclerosis; and 4) there are inherent limitations to any meta-analysis based on published articles, including the fact that the analysis was not based on individual patient data and the inevitable publication bias.

## CONCLUSION

This study found that alcohol intake was not associated with the risk of multiple sclerosis, whereas beer intake was associated with an increased risk of multiple sclerosis. Moreover, the role of alcohol intake on the risk of multiple sclerosis might differ between males and females, which needs further verification through a large-scale prospective cohort study.
